# Selections and Abstracts

**Published:** 1900-06

**Authors:** 


### *SELECTIONS AND ABSTRACTS.*

---

### What Vermouth is Made Of.

It is by no means a matter for congratulation, says *Food and Sanitation*, that the practice of taking "appetizers" has greatly increased in England during the past few years, introduced mainly through that brutalized class of our population, the members of the Stock Exchange. The disgraceful spectacle afforded by the élite of our legalized "gambling hell" some days ago, when some fifty of its blackguards knocked down and kicked *one* foreign member, and when he was stunned, bruised and helpless, again brutally assaulted him, shows plainly the price the *morale* of the English people is paying to-day for its worship of the Barnatos and like scum of the earth.

When the observer—hating politics, big endians, and little endians, alike—reflects that it is for this class of cowardly rogues and their craven congeners cowering in Cape Town—"Outlanders," who take good care to be "out" of the way when brave English, Irish, Scotch, and Welshmen are fighting for England's honor—that we are now at war, our prestige at stake, and the certainty before us of taxes at a ruinous rate, with bad trade, and the evils that follow in its train, it may be worth while to study somewhat this question of "Appetizers."

It is a curious coincidence that it was the Methuen Treaty of 1703 which caused John Home to write:

> "Firm and erect the Caledonian stood,
> Prime was his mutton and his claret good;
> Let him drink port, an English statesman cried,
> He drank the poison, and his spirit died!"

And that it should be whilst smarting under the present Lord Methuen's disaster the "appetizer" imbibing cads of our Stock Exchange should have shown their quality by brutally assaulting *en masse* one helpless man. This is not, however, surprising when one examines the kind of poisonous mixture the blackguards swallow. One French vermouth, now very much in fashion amongst them, contains on an average one-ninth of its bulk of 85 per cent. alcohol. The following ingredients enter into its composition:

Dry white wine, muscatel wine, bitter orange peel, wormwood, chamomile, water germander, florentine iris root, centaury, peruvian bark, aloes, cinnamon, nutmeg, raspberry juice, fish glue, helenium, calamus odoratus, holy thistle, angelica root, gentian.

It would be hard to imagine anything better arranged than this to develop and bring to the surface every brutal instinct a man may possess. It nearly equals the celebrated "Alexander's Golden Antidote," "a sample," as Mark Twain says, "of the earthquakes which the old-time doctor used to introduce into his patient when he could find room. It is good for pretty nearly everything." It was probably the old original first patent medicine, which was built as follows:

"Take of afarabocca, henbane, carpobalsamum, each two drams and a half; of cloves, opium, myrrh, cyperus, each two drams; of opobalsamum, Indian leaf, cinnamon, zedoary, ginger, coftus, coral, cassia euphorbium, gum tragacanth, frankincense, styrax calamita, celtic, nard, spignel, hartwort, mustard, saxifrage, dill, anise, each one dram; of xylaloes, rheum, ponticum, alipta moschata, castor, spikenhard, galangals, opoponax, anacardium, mastich, brimstone, peony, eringo, pulp of dates, red and white hermodactyls, roses, thyme, acorns, pennyroyal, gentian, the bark of the root of mandrake, germander, valarian, bishops weed, bay berries, long and white pepper, xylobalsamum, carnabadium, macodonian, parsley seeds, lovage, the seeds of rue and sinon, of each a dram; of pure gold, pure silver, pearls not perforated, the blatta byzantina, the bone of the stag's heart, of each the quantity of fourteen grains of wheat; of sapphire, emerald, and jasper stones, each one dram; of hasle nut, two drams; of pellitory of Spain, shavings of ivory, calamus odoratus, each the quantity of twenty-nine grains of wheat; of honey and sugar, a sufficient quantity." "Serve with a shovel?" the great humanitarian, Mark Twain, asks. "No, one might expect such an injunction after such formidable preparations; but it is not so. The dose recommended is the quantity of a hazelnut, only that; it is because there is so much jewely in it, no doubt.

For the health of its consumers it is a pity there is no "pure gold, pure silver, pearls not perforated, sapphire, emerald, and jasper stones" in vermouth. If there were, the drinking of this abomination would not be spread broadcast by our Stock Exchange cads. They would not drink it, but pocket it.—*Dietetic and Hygienic Gazette.*

---

### THE METRIC SYSTEM AGAIN.

The *Philadelphia Medical Journal* has lately contained some discussion of the metric system, and one or two correspondents have indorsed "the eminently practical method," long advocated with some modification of detail, which depends on thinking in grains, writing in grams and ordering the total to be divided into fifteen equal parts. It seems to us that such a method, instead of being eminently practical, is simply an example of fetichism. We are reminded of the manufacturing pharmacist who a year or two ago littered some parts of the country with circulars descriptive of "Oleum Morrhua Compositus," evidently thinking there was some mysterious efficacy in Latin, even if it was incorrect. The person who essays to learn a foreign language by the literal translation of one word into another, ignoring grammatic construction, idioms and pronunciation, has gone through a great deal of mental drudgery and has practically nothing to show for his pains. The physician who thinks in grains, writes in grams, and virtually requires the druggist to think backward for him, puts himself to considerable trouble for nothing.

The scientific advantages of exact correspondence between units of weight, volume and length possessed by the metric system are of little practical use in prescription-writing, nor is there any virtue in the metric system, in and of itself. From a personal experience of ten years with the metric system, after a previous use of the apothecary's, we are prepared to vouch for the simplicity and convenience of the decimal system. But we can very readily imagine that older and more conservative men might, from long custom, use the apothecary's weights and measures to better advantage, just as pounds, shillings and pence persisted for some years after the adoption of our decimal monetary system. "The eminently practical method" advocated in our esteemed contemporary's columns is an excellent compromise for such as are too old to learn the metric system and yet wish to give it their support, but we object to the futility of teaching this method to students. If the decimal system is not adapted to the needs of those not hampered by familiarity with the lack of system pertaining to older tables of denominate numbers, the sooner it is dropped the better.

The best and simplest way for any one not familiar with the metric system to acquire facility in its use is to study dose-values. The milligram represents the approximate dose of drugs of the highest toxicity, such as atropine. There is probably no chemic substance, unless the organic toxins, which could not be given with safety in this dose to a healthy adult, and only a very few, like curarin and hyoscine, whose average, standard, adult dose is less than a milligram. The milder alkaloids, such as morphine, pilocarpine and cocain, range about the centigram. The most poisonous metals and metaloids, such as arsenic, phosphorus and mercuricum, intermediate alkaloids like strychnine, etc., lie between the milligram and the centigram. Comparatively non-toxic metals, such as bismuth, sodium and potassium, and the milder synthetic nervines range about the gram. Saline and oleaginous cathartics are usually given in from 10 to 20 gram doses.

In writing prescriptions there can be no confusion if we remember that the gram (including the cubic centimeter or fluid gram) corresponds to the dollar, the centigram to the cent, the milligram to the mill, and use the decimal point or line precisely as in making a statement of an account. It is simpler and safer to order for pills, suppositories and all other separate pharmaceutic preparations, the amount of each ingredient for each piece, directing the druggist that "Fiant pululæ (capsulæ, suppositoria, etc.) tales No. x (xx, etc.)."

For liquid prescriptions for external use we simply write in percentages, remembering that 1000 c.c. corresponds to a quart. For ointments the amount will vary from 25 to 100 grams, according to the surface to be covered. Internal prescriptions are commonly measured in teaspoonfuls, an old, worn spoon holding about 4 c.c., a new one 5 c.c., or in tablespoonfuls, containing 15 to 20 c.c., according as the spoon is level or heaped. Thus, the entire prescription will be for 10, 20, 30, etc., times the dose-measure, and each ingredient will be multiplied in the same manner.—*American Therapist.*

---

### X-RAY DIAGNOSIS OF BILIARY AND RENAL CALCULI.

Until recently the use of the radiograph has been considered unsatisfactory in cases of renal calculi, and useless in detecting gallstones. Of late, however, considerable improvements in technique have been made.

Leonard, in the *Philadelphia Medical Journal* of January 6, 1900, and the *Annals of Surgery*, February, 1900, has discussed the subject of renal calculi very exhaustively. He has shown that the principal difficulty encountered is that the calculi are very little more opaque than the tissues of the abdomen, and consequently do not show in the negative taken. Leonard's method "is founded on the axiom that if rays are employed that will differentiate between the shadows of the tissues that are less dense than the least dense calculus all calculi will be found. Thus, in examining for calculi we do not want rays that will penetrate all tissue, but a differentiation in shadows that will demonstrate without doubt that all calculi, no matter what their relative density, will cast shadows if present in the field of examination. Such differentiation makes the negative diagnosis absolute. The result sought for in examining for renal calculi is to obtain negatives in which shadows are shown of the tissues less opaque than the least opaque calculi."

Experience has demonstrated the fact that this tissue differentiation can be obtained by employing a large volume of Röntgen discharge from a tube of low vacuum, or soft tube, as it is called. For this purpose a self-regulating tube is the most satisfactory. Using these methods, Leonard has succeeded in detecting calculi in the kidney and ureter twelve times out of fifty-nine cases in which the symptoms were either very marked, or uncertain, or where a negative diagnosis needed confirmation. In eight of the twelve cases so diagnosticated the presence of calculi was confirmed by operation. Seven cases in which a negative diagnosis had been made were subsequently operated upon, and no stone was found, with the exception of one case where the error was due to faulty technique. Of particular interest is the fact that Leonard has succeeded three times in locating small calculi in the lower end of the ureter, there being only one other case on record where this has been done. A careful review of the literature shows that renal or ureteral calculi have been successfully located by the x-ray in thirty-six cases by many different observers, so that the possibility of detecting such calculi by this means may be considered to be established, and thus a very important diagnostic aid given to surgery. The absolute localization of the calculi and the precise knowledge of the number of calculi materially facilitates and simplifies operative procedure and precludes incomplete operation. The absolute negative diagnosis renders non-operative treatment rational, when its omission might otherwise endanger the functional activity of either or both kidneys, or even the patient's life.

Leonard thinks that the positive and negative diagnosis are rendered absolute by the present technique, but that the operator must be able to show in his negative differentiation shadows of the less dense tissues in the lumbar and pelvic regions and that one perfect negative, showing such detail, is sufficient evidence on which to pass a negative diagnosis.—*Maryland Medical Journal.*

---

### THE TREATMENT OF MENORRHAGIA IN YOUNG GIRLS.

The treatment will depend upon the pathologic condition which causes the hemorrhage, and it is rare that one is compelled to treat the bleeding directly. In case of extreme necessity, however, we may use very hot douches; simple or with gelatin solution, small quantities of fluid frequently repeated (200 c.c.). Rectal irrigations with very hot water by means of the double current tube may be used for a considerable length of time. The application of ice to the abdomen, the administration of ergotin by mouth, by rectum, or hypodermatically are measures which will seldom fail to arrest the bleeding. Above all, complete rest in bed is an essential part of the treatment.

When these girls come to the physician on account of an immoderate flow of blood at each menstrual period, they must be put to bed during their menses and not allowed to get up for twenty-four to forty-eight hours after all signs of bleeding have disappeared. They must remain as quiet as possible, and not even rise in bed for their meals. At the onset of each period the treatment must be renewed, and it may be said that the rest in bed is more difficult to enforce than any other measure, be it ever so disagreeable. It will be found, however, that as the treatment progresses there will be a shortening of the duration of the menstrual period and a diminution of the amount of blood lost. When girls are allowed to get up they must be cautioned against too great physical or mental exertion of any kind, and all such forms of exercise as bicycling, riding, sewing on the machine, prolonged walking, dancing and running must be for the time being prohibited. Later these may be allowed in moderation, and their effects upon the genital apparatus carefully watched.

The prophylactic treatment consists in getting the body into the best possible condition. This means proper hygiene, especially as to diet, exercise, clothing, bathing, etc. Cold sponging and frictions will help to restore to the skin its eliminative functions. Cold douches are excellent stimulants to the nervous and circulatory system, and next to rest in bed, the most efficient measure. Baths tend to bring on the menstrual flow, and must therefore be taken lukewarm rather than cold, and only be of short duration. Spices, game, preserved meats, alcoholic beverages, and tea and coffee in excessive quantity must be avoided. Fresh air, especially a sojourn in the country, if possible in the mountains rather than at the seashore, is very beneficial. We must not wait until anemia sets in, for then the treatment is more difficult.—*Pediatrics.* (*Reprinted from the Journal des Fracticiens, December 16, 1899. By Armand Siredey.*)

---

### TYPHOID FEVER IN THE YOUNG.

A Jacobi in *Pediatrics* of December 15, 1899, advises the study of the older works, such as Taupin's Relliet's and Edmund Fuedrich's. The opportunities for infection are the same in the young as in the adult. Water is usually the medium, and the fact that it is generally boiled explains the relative absence of typhoid in the first years of life The severity of the illness does not correspond with body temperature, the worst cases of sepsis sometimes exhibiting the lowest temperature. The rise in temperature is mostly gradual. It is high in the second stage, and frequently irregular, owing to complications, such as otitis media, scarlatina, malaria or suppurating arthritis, or to the nature of the individual infection. The lips, tongue and mouth exhibit the same surface changes of the epithelium as in the adult. Vomiting is noticed in bad cases. Diarrhea of a catarrhal nature, depending on the presence of toxins or of ulcerations, is a frequent symptom. Intestinal ulcerations are not invariably met with, and when present do not necessarily cause diarrhea. Constipation is a not uncommon symptom in the first as well as the second week. Gurgling in the ileocecal region is common in intestinal catarrh, and is not characteristic of typhoid fever. Tympanitis is usually moderate, extensive peritonitis being uncommon. The local form is, however, quite frequent. Hemorrhages are very rare, and are mild in children over four years old. The organs of circulation are not affected to the same extent as in adults. If the pulse becomes feeble and frequent owing to toxic and organic myocardial weakness, persistent stimulation should be resorted to. A decrease in the size of the spleen during the third week is a good symptom. Permanence in the swelling is infrequent and abscesses rare. The nasal mucous membrane is dry and irritated. Epistaxis occurs in older children. A catarrhal condition or ulceration of the pharynx and larynx may cause hoarseness and cough, or dyspnea and strangulation. Bronchial catarrh is frequent; pneumonia, either catarrhal or hypostatic, may occur; pleuritis and diphtheria are rare. The urine is of high color; urea and uric acid are increased, and chlorides decreased. Indican, albumin, renal epithelium and casts frequently are present. The Diazo test is usually positive from the end of the first week to the middle of the third. The skin is usually dry. The characteristic roseola is the same as in the adult. Miliara and erythema, gangrene, abscess, furuncles and pustules occur. Desquamation may be copious. Periostitis and osteomyelitis are uncommon. The nervous system is not affected as it is in adults.

Ambulant cases are common. Headache is frequently complained of; grinding of the teeth, sopor or delirium, and screams resembling those of meningitis are met with. Aphasia sometimes occurs; polyneuritis is not rare; hemiplegia is rare; paraplegia more frequent. Mania and melancholia are the forms of psychical disturbance most frequently seen. Typhoid fever may occur in the fetus; it is rare in earliest infancy; more common in the second and third years, and after that time increasing rapidly in frequency. Different authorities differ widely as to the years of greatest frequency and mortality. The diagnosis is sometimes difficult, the Widal and Diazo tests being important aids. Meningitis may be recogized by Kernig's symptom or examination of the cerebro-spinal fluid. The mortality varies according to season and the children, and ranges from two to nine per cent., the very young and adolescents being more likely to succumb. Complications are dangerous; relapses not rare. The food should be liquid, and water should be given frequently. A purgative dose of calomel at the beginning, and warm water enemata later in the disease act beneficially. For offensive diarrhea, permanganate potassium or thymol enemata; internally, bismuth, sulpho-carbolate of zinc, salol and naphthalin. Collapse requires stimulants by mouth and subcutaneously. Chloral hydrate is the best hypnotic. Sopor or coma should be treated with cold affusions, while the body is submerged in water of 90 to 95°. Antipyrin is safer than acetanilid or salicylates. Cold and warm water are the safest antipyretics. As cold water is often contraindicated the author advises the use of warm baths, 90 to 95°, accompanied by friction, every three or four hours throughout the illness.—*Medical Fortnightly.*

---

### SLEEPLESSNESS.

Sir William H. Broadbent treats this subject in a practical manner in *The Lancet* of January 27, 1900. There are some instances in which it is wise to resort to opiates or sedatives, but as a rule, he condemns them. When the brain has been taxed by engrossing work, or the nervous system shattered by a severe shock, exhausted by anxiety, worn out by excitement, or when one's habit of sleep has been broken by watching the sick, a judiciously selected remedy may restore the normal conditions, and regular habits may be regained by its limited use.

Some people are naturally bad sleepers; the merest trifle will keep them awake, and these are to be pitied. Even with these he hesitates to give drugs, especially if they can lie quietly and rest. In such cases the bromids should be employed, if tolerated; there are cases in which moderate doses of bromid readjust the irritable nervous system, and their long use seems to produce no bad effects.

Anemic girls are often kept awake by cold feet. For these the hot bottle, or enveloping the feet and legs up to and above the knees in warm flannel are simple means of overcoming the difficulty. These patients should prepare for bed quickly, and their feet should be vigorously rubbed before they try to sleep. A little very hot and strong beef-tea or hot milk will have a very soothing and warming effect, but stimulants should be avoided in young persons. For this condition in brain-workers, heat applied externally seems to have no beneficial effect. Hot beef-tea, hot milk, or even hot water is especially valuable in these cases, but the chief resource is friction. Standing in cold water for a few minutes and then briskly rubbing the feet has proved particularly effectual.

Certain conditions of the circulation often give rise to sleeplessness. One of these is high arterial tension. The patient's diet must be regulated, but changes from customary habits must not be too radical. A glass of water night and morning is usually good, and a mild mercurial aperient is efficacious, continued once or twice a week for some time.

The most common cause of sleeplessness is indigestion in various forms, especially that causing flatulence. He thinks this is due to the mechanical pressure or stretching, which prevents sleep. A glass of hot water before retiring will usually relieve, but the dyspepsia which causes the disturbance should receive medical treatment. The hot water should be taken before undressing, that the gas may have time to escape before the patient lies down. Should this not prove beneficial, sal volatile and carbonate of soda may be taken before it, or an alkaline draught may be given—carbonate and sulphocarbolate of soda with aromatic spirit of ammonia, compound tincture of chloroform or ether, and peppermint or camphor water, and sometimes bromid of sodium or ammonium may be added with advantage for a time. Friction over the epigastrum or between the shoulders may help to disburse the flatulence. He does not believe in allowing the taking of even these simple medicaments to become a habit.

Sleeplesness following influenza should be treated as an acute affection; arsenic or phosphorus, strychnin and quinin, with measures for the relief of functional derangements, should be resorted to. Should these not prove effectual, opiates may be administered without hesitation. He thinks it better to resort at once to combinations of opium or morphine and hyoscyamus with carminatives, than to try sulphonal, trional, chloral or bromids. Morphine should be administered hypodermically, and it is well to combine it with strychnine as well as atropine. The sleeplessness following prolonged use of alcohol, which may end in delirium tremens, should be treated by the withdrawal of all alcoholics and considerable doses of strychnine or nux vomica, and possibly digitalis. The liver and stomach derangements from the alcohol should have prompt attention, as they may keep up the sleeplessness, being possibly its main cause.—*Doctor's Magazine.*

---

### PROGRESSIVE PERNICIOUS ANEMIA AND MALIGNANT DISEASE OF THE STOMACH, AND THEIR TREATMENT.

Dr. A. Abrams (*Medical Record,* April 28, 1900) says that arsenic is a true specific in pernicious anemia, and is as certain in its immediate results as is mercury in syphilis, quinine in malaria, or iron in chlorosis. The specificity of arsenic is so great that in no case of grave anemia are we justified in excluding the progressive pernicious variety, even though the blood examination is negative, without a heroic trial of arsenic. Like the other specifics, it produces relative cures, and cannot be regarded as a prophylactic, owing to the frequent relapses which occur. It may be given as Fowler's solution, beginning with three-minim doses, well diluted, after each meal, and increased by one or two minims daily, according to the urgency of the case, until twenty-five or thirty minims is taken three times a day. A safer rule is to push it to the point of toleration and maintain it at this point until the blood examination shows the result desired. The appearance of its physiological effects (edema and itching of the eyelids, gastro-intestinal irritation, etc.) is a signal for its temporary discontinuance. When arsenic cannot be given by the stomach, it may be administered subcutaneously or even by the rectum.

In association with arsenic, assimilable food and rest are indispensable adjuvants.

The use of intestinal antiseptics in this as well as in other diseases is a mere therapeutic refinement not sanctioned by bacteriological reasons, and they ought not, therefore, to be employed as a routine measure. In the Italian literature one finds some authentic evidence of the good effects from thymol, its administration being suggested by the theory that pernicious anemia is caused by intestinal absorption of products which are destructive to the red blood-corpuscles.

Iron is not only useless but is apt to create digestive disturbances.

Bone-marrow is said to be curative, but in my experience it induces nausea and aggravates existing gastro-intestinal troubles.

Gastric disturbances suggest stomach lavage. The character of the food ingested must be determined by the results of a chemical analysis of the stomach contents.

To counteract the great reduction in the quantity of blood (oligemia) weak saline solution may be given by the colon (enteroclysis) or preferably in the subcutaneous tissue (hypodermoclysis).

Relapses are best prevented by minute attention to dietetic and hygienic details.—*Medical Age.*

---

### THE BUBONIC PLAGUE.

After so long a term without a visit from the plague, our firm conviction of natural freedom has received a profound shock by the recent appearance of this disease almost simultaneously at both of our seaboards and in our insular possessions. With casual interest we observed from afar its ravages in India, China and other distant Eastern countries, and the recent outbreaks in Portugal and Brazil opened our eyes to a possible encounter; but not until a ship from South America and another from Japan brought us this dread visitant was the American public made fully aware of our danger. While we often have types of this disease that are unmistakable too frequently it assumes bizarre forms, with fever, as Yearsin says, as the only constant symptom, and if of a mild type buboes may be so slight as to escape detection, or on the other hand it may assume the pneumonia type with no characteristic signs, but from this same mild form a most virulent epidemic may start, as the experience in Honolulu and Portugal showed too well. The outbreak in San Francisco seems to have been nipped in the bud, thanks to vigorous means; but if it had once gained headway in Chinatown the consequences would have been disastrous. The small outbreak in Honolulu, at first diagnosed as beri-beri, has cost us several millions of dollars in a few months, but seems now to be effectually stopped. Not so long ago we learned from Backer that as far as he could learn the bubonic pest had never been known in the Philippines; but hardly before we had finished our self-congratulations, reports reached us of an outbreak that is still uncertain in its results. Sanitary conditions in many of our coast towns are ripe for a speedy propagation of this dreadful pest. True, we have sera and vaccines to cure or protect when once the malady is recognized; but we cannot rely too much on what is still in the formative stage.

An ounce of prevention is worth a pound of cure, and we can well feel proud of our efficient quarantine system; but with this black pest knocking at our very doors, we must also consider the many improvements that might beneficently be introduced. The whole system should be put into the Marine Service hands, and we then need expect no such clash as was recently seen in New York between the government and municipal authorities. Abroad, at every port should be stationed a medical officer who understands the infectious diseases, who understands the people and their language, and who is clothed with power to delay embarkation of suspects to this country. At present we do control, through our bill-of-health rules, all of the vessels bearing imports to the United States ports if they come from large centers, but the system should be extended to minor points, and consulships and marine appointments should be combined in the same officer at little increased expense, and by his weekly telegraph report to the surgeon-general no suspects could sail or infected vessels land without a full knowledge in Washington and subsequent compulsory quarantine. Let the door be locked before the horse is stolen.—*Med. Review.*

---

### AMPUTATION AND LONGEVITY.

A prominent manufacturer of artificial limbs says (*Popular Science News*): "By looking over books which comprise the history of many thousand cripples, we arrive at the conclusion that dismembering plays no part whatever in shortening life. Our records date back to 1853, and it is an astonishing fact that, of the entire number of our patrons, less than twenty-five per cent. have died, and most of those from old age or accident; and in no case can we learn of a death that can be directly ascribed to the loss of a limb. As we investigate this subject more thoroughly we are persuaded that amputations enhance vitality, render it not only probable, but positive, that on account of amputations the lives of the subjects will be prolonged and free from disease." No record can be found of a cripple becoming insane, and but very few cases where they have committed suicide. The mental as well as the vital forces appear to become strengthened by the dismemberment.

It is a noticeable fact that persons who lose their legs become very powerful in their arms, large in chest, and great in girth, and persons who lose their arms become powerful in their legs and large in girth. The loss of parts of the body conduces to health, life, and development. Dare, Melrose, Conway, Leland, and Fitzpatrick, one-legged acrobats whose muscular developments are the envy of the world, have never been surpassed by athletes with natural limbs.

A reasonable explanation may be found in the hypothesis that the removal of a part of the body lessens the demand on the vital forces, and permits the supplying reservoirs to contribute more abundantly to the remaining members. If it overtaxes the heart to force the blood through all the avenues of the body, will not its labors be lessened if a few of those avenues are forever removed? And will not the remaining avenues receive a larger proportion of the life-giving essences? If the nervous system is overburdened, will not the tax be lessened if a part of the nerve organization be removed? If a tree is permitted to grow, it will sap itself by the many choking branches that come from its trunk. The cutting off of these branches and the trimming up of the limbs always give new vigor to the tree. It will grow larger, stronger, and will live longer. A cripple is neither a cynic nor a pessimist. His misfortune has driven from him whatever there may have been of the misanthropic. His health is always good, and he is therefore happier and more contented than the dyspeptic or rheumatic. Nature, with her usual generosity, compensates for every misfortune. Those who seem the most afflicted are often happiest; their minds have been prepared to endure misfortune.—*Medical Age.*

---

### INSIDE INFORMATION.

It is now proposed to take photographs of the interior of the stomach by means of an instrument, devised by two scientists with unpronounceable names, consisting of a series of lenses attached to a stomach tube containing an electric light. We hail this discovery with unfeigned joy. In cases of disputes at lunch counters as to whether the eater partook of pork and beans at ten cents per dish or ham and eggs at twice that amount, it will only be necessary for the customer to indulge in the trifling feat of swallowing a pocket kodak attached to a string and the results may be had in short order.

Fond mothers can ascertain the value to be attached to the youngest son's protestations of innocence regarding the disappearance of sundry cakes and pies. Photographs of tough steak bravely resisting the onslaught of gastric juice can be shown to the hardened wretch who vends horse-sausage and cuts porterhouse steaks off a choice part of the back of the ancient bovine neck. On the other hand the dyspepsia cure men can, and doubtless will, exhibit scenes in which are depicted the mighty dyspepsia tablet pulverizing and dissolving india-rubber, oyster cans, old shoes, and even the perennial spring chicken, although the latter involves quite a feat of imagination. These things will melt away before the ordinary combination of white sugar and pepsin as does ice-cream melt in the vicinity of the summer maiden.

Even so will the instrument be of much avail in fixing the guilt of the wily Senegambian during watermelon time. Not only will the bulging of the lower part of the vest (if he wears one) be strong circumstantial evidence, but the photograph of the interior of Africa will show where the guilt and the watermelon lie, and also the Senegambian.—*The New Idea.*

---

**This Journal and Atlanta Semi-weekly Journal one year for $1.00 cash in advance. No checks. Strictly to new subscribers.**

---

### THE LAST WORDS OF MEIAMOUN.

To a lover of the exquisite in literature there is nothing more beautiful than that which was given the literary world by Theophile Gautier, and we could wish that he had written more voluminously. Whether or not his writings are obscene depends on the breadth of view of the reader. It seems to us that the seeker after the questionable would hardly look for it in such artistic gems. However this may be, Dr. C. A. L. Reed, following the style of Gautier, wrote under the above title. His prose-poem is so like those of Gautier that had it been published in connection with "One of Cleopatra's Nights," it would have been considered a part of the original. This was published in the *Medical Mirror* of June, 1899. The sequel comes on the 15th of May, 1900, when the postal authorities called on Dr. Love to convince them that it was mailable matter. There is doubtless medical politics behind the complaint, and it is reasonably certain that the right of the medical press to liberty beyond that granted to journals destined for the perusal of children will be demonstrated. The question distinctly concerns every medical journalist. None of us wish to cater to hunters for obscenity, or to devote our pages to what is questionable, but we do wish that the intelligence and standing of our readers and contributors be considered, and that we be judged on that basis. The claim that a medical journal may publish only scientific matter is also to be met at this time, and the decision in Dr. Love's case will determine the opinion of the department on the question. It should be the personal business of every medical journalist that this feature of the question be answered decisively, that all sides of the physician's intellectual life may be served through this medium.—*Medical Fortnightly.*

---

**This Journal and Atlanta Semi-weekly Journal one year for $1.00 cash in advance. No checks. Strictly to new subscribers.**

---

### THE WAY TO EMPLOY COLD IN TYPHOID FEVER.

Johnson (*Medical Times*) says that in private practice the objections of friends and the difficulty of procuring a suitable tub and capable assistant often make it inexpedient to use the tub treatment. In these cases cold sponging may be used. If the temperature is high and obstinate, the patient should be stripped and the bed protected by a rubber blanket, over which is placed a sheet. Then the nurse takes a piece of ice and sweeps it over the body vigorously, at the same time applying friction with the other hand, or having this done by another nurse. This friction is a most important part of the procedure, it increasing the loss of heat by at least fifty per cent. over the application of cold alone. Pressure should not be great, but active friction with the ball of the thumb and palm of the hand until the skin is red from the reaction. If friction cannot be used, then do not apply cold, as the two must go together. Patients who object to hydrotherapy can often be induced to yield to its employment by beginning gradually. First a tepid sponging may be given, and gradually less tepid water may be used until cold water is employed. Then state to the patient, or friends, that the point in the disease has been reached when water with pieces of ice in it is needed, and finally ice-water or pieces of ice, as described, may be used, when it is found that the patient does not catch cold, as it is always feared will be the case. Frequently people will think it all right if alcohol is added to the water. This may be done, as it also adds to the cooling properties of the sponging.

---

**This Journal and Atlanta Semi-weekly Journal one year for $1.00 cash in advance. No checks. Strictly to new subscribers.**

---

### LACTATION BY A VIRGIN.

Now and then we come across tales of men having performed the function of lactation, and everybody regards them as wonderful, if true. But little less astonishing is an account (*Médecin Moderne*, November 15, 1899; *Lyon Médical*, December 3, 1899) of the experience of a young woman who had never had sexual intercourse. She undertook to care for a six-months-old infant during the daytime. It refused to take milk from a glass, so, to soothe it, she gave it the breast. Getting nothing to repay its efforts, the child became angry. The attempt was repeated several times during the day, and at night the child was nursed by its mother. The young woman persisted in her attempts, and she felt a pleasurable sensation while the child was sucking. After a time she found that her breasts had grown larger, and at the same time she realized that the child was keeping his hold of the nipple longer and longer and was getting milk. Eventually her milk became so abundant as to plague her; the breasts were so distended at night that her sleep was interfered with, and she longed for morning to come that the child might relieve her. When by chance it was kept away from her she suffered severely. She seems to have known nothing of the breast-pump.—*N. Y. Medical Journal.*

---

### WHY I USE PEPTO-MANGAN "GUDE."—AN EXPERIMENTAL DEMONSTRATION.*

BY WM. KRAUSS, PH. G., M.D., Memphis, Director of the Microscopic Laboratories, Memphis Medical College; Pathologist and Visiting Physician to St. Joseph's Hospital, etc., etc.

Some five years ago I wrote a paper for the *Memphis Medical Monthly*, giving a résumé of the evolution of the iron compounds,

* Read before the Memphis Medical Society.

and appended a report of cases giving blood counts, etc. The manufacturers of the preparation I preferred saw fit to reproduce the case reports in their pamphlets, but said nothing about the reasons that induced me to prefer their product.

At a recent joint meeting of physicians and pharmacists I was criticized for opposing the use of ready-made compounds, while still advocating the use of Pepto-Mangan "Gude," which is a proprietary preparation. I hesitated considerably about bringing the matter up again, because I dislike to build up a reputation as an indorser, *and have never in any other instance written an article indorsing a proprietary preparation.*

I hope, however, to show you this evening that there is no pharmacopœial preparation that meets the requirements of an ideal iron compound, and, until this is found, I intend to continue to use what has never disappointed me, and is not based upon mere faith. The work of Bunge is too well known to be now quoted, and I will only make a few experiments before you this evening and show you the reasons for the faith that is in me. There may be other proprietary iron compounds, and doubtless there are, that will come up to the same requirements, but I see no advantage in swapping the devil for the witch.

It is not necessary to repeat all the tests with all the official iron preparations, because they are divisable into groups, all the salts of one group behaving very much alike toward the gastric and intestinal juices.

An ingenious theory recently put forward regarding the action of the mineral salts of iron is, that they decompose the substances in the intestinal tract which precipitate the *food iron* so that it may be absorbed. That is the only rational explanation of the fact that we do occasionally get results from them. On the other hand, it is far more rational to use an iron compound that can be, and is absorbed, for then we are reckoning with known quantities, instead of blundering along, giving more iron at a dose than is contained in the entire body, and incidentally deranging the digestive functions by precipitating the gastric, pancreatic and intestinal juices, and producing constipation by reason of the very astringent nature of some of the iron salts.

Beginning with the organic double salts, of which the scale salts are representatives, we notice upon the addition of this gastric juice, that a precipitate is formed; the double salt is decomposed and ferric salt remains, which is insoluble, both in gastric and intestinal juice.

The tincture of ferric chlorid will precipitate some of the gastric constituents, though most of the iron will remain in solution in the hydrochloric acid; the iron still in solution will not be absorbed, because its non-diffusibility is taken advantage of in the manufacture of *dialised iron*, the acid passing through the animal membrane; when the iron finally reaches the intestine, the alkalin carbonates promptly precipitate it. Ferrous sulfate behaves similarly. In both instances, as you see, the very insoluble ferric oxid is finally formed. If you have ever tried to remove iron stains from your water pitcher, you have some idea how insoluble it is.

The insoluble compounds, like reduced iron, or Vallet's mass, only serve to render inert the arsenic with which they are usually prescribed; if dissolved at all in the stomach, they are re-precipitated in the intestine.

Taking now Gude's preparation, we find it soluble, not only in all these reagents, but also in a mixture of them. Potassium ferrocyanid readily gives the iron reaction, excess of ammonia will separate it, redissolving the manganese, which is then recognized by the color of its sulfid; the alkalin copper solution gives the reaction for pepton, showing that it is what the label says. It mixes with arsenious acid, forming a perfect solution, thus giving us a most useful hematopoietic agent. The soluble alkaloids are perfectly soluble in it, as is also mercuric chlorid. Being a pepton, it is readily diffusible by osmosis.

The only disturbing agent in the intestinal tract is hydrogen sulfid; this will precipitate it, but, presumably, much of the iron must have been absorbed before it encounters this gas; if not, appropriate agents should be used for its elimination.

Therapeutically, it does not nauseate, constipate, discolor the teeth, precipitate the digestive agents, nor become inert from contact with them. As to the clinical results, I need not add anything to the many reports already on record.

*210 to 220 Randolph Building.*

